# Study of CD27, CD38, HLA-DR and Ki-67 immune profiles for the characterization of active tuberculosis, latent infection and end of treatment

**DOI:** 10.3389/fmicb.2022.885312

**Published:** 2022-07-22

**Authors:** Sergio Díaz-Fernández, Raquel Villar-Hernández, Zoran Stojanovic, Marco Fernández, Maria Luiza De Souza Galvão, Guillermo Tolosa, Adrián Sánchez-Montalva, Jorge Abad, María Ángeles Jiménez-Fuentes, Guillem Safont, Iris Romero, Josefina Sabrià, Cristina Prat, Jose Domínguez, Irene Latorre

**Affiliations:** ^1^Institut d’Investigació Germans Trias i Pujol, Barcelona, Spain; ^2^CIBER Enfermedades Respiratorias, CIBERES, Instituto de Salud Carlos III, Madrid, Spain; ^3^Departament de Genètica i Microbiologia, Universitat Autònoma de Barcelona, Barcelona, Spain; ^4^Servei de Pneumologia, Hospital Universitari Germans Trias i Pujol, Barcelona, Spain; ^5^Plataforma de Citometría, Institut d’Investigació Germans Trias i Pujol, Barcelona, Spain; ^6^Unitat de Tuberculosi de Drassanes, Hospital Universitari Vall d’Hebron, Barcelona, Spain; ^7^Universidad de la Frontera (UFRO), Temuco, Chile; ^8^Infectious Diseases Department, Vall d’Hebron University Hospital, PROSICS Barcelona, Universitat Autònoma de Barcelona, Barcelona, Spain; ^9^Grupo de Estudio de micobacterias (GEIM), Sociedad Española de Enfermedades Infecciosas y Microbiología Clínica (SEIMC), Madrid, Spain; ^10^Hospital de Sant Joan Despí Moisès Broggi, Barcelona, Spain; ^11^Julius Center for Health Sciences and Primary Care, University Medical Center Utrecht, Utrecht University, Utrecht, Netherlands

**Keywords:** *Mycobacterium tuberculosis*, T-cells, immune response, activation markers, treatment, flow cytometry, multiparametric analysis, cluster

## Abstract

**Background:**

Current blood-based diagnostic tools for TB are insufficient to properly characterize the distinct stages of TB, from the latent infection (LTBI) to its active form (aTB); nor can they assess treatment efficacy. Several immune cell biomarkers have been proposed as potential candidates for the development of improved diagnostic tools.

**Objective:**

To compare the capacity of CD27, HLA-DR, CD38 and Ki-67 markers to characterize LTBI, active TB and patients who ended treatment and resolved TB.

**Methods:**

Blood was collected from 45 patients defined according to clinical and microbiological criteria as: LTBI, aTB with less than 1 month of treatment and aTB after completing treatment. Peripheral blood mononuclear cells were stimulated with ESAT-6/CFP-10 or PPD antigens and acquired for flow cytometry after labelling with conjugated antibodies against CD3, CD4, CD8, CD27, IFN-γ, TNF-α, CD38, HLA-DR, and Ki-67. Conventional and multiparametric analyses were done with FlowJo and OMIQ, respectively.

**Results:**

The expression of CD27, CD38, HLA-DR and Ki-67 markers was analyzed in CD4^+^ T-cells producing IFN-γ and/or TNF-α cytokines after ESAT-6/CFP-10 or PPD stimulation. Within antigen-responsive CD4^+^ T-cells, CD27^−^ and CD38^+^ (ESAT-6/CFP-10-specific), and HLA-DR^+^ and Ki-67^+^ (PPD- and ESAT-6/CFP-10-specific) populations were significantly increased in aTB compared to LTBI. Ki-67 demonstrated the best discriminative performance as evaluated by ROC analyses (AUC > 0.9 after PPD stimulation). Data also points to a significant change in the expression of CD38 (ESAT-6/CFP-10-specific) and Ki-67 (PPD- and ESAT-6/CFP-10-specific) after ending the anti-TB treatment regimen. Furthermore, ratio based on the CD27 median fluorescence intensity in CD4^+^ T-cells over *Mtb*-specific CD4^+^ T-cells showed a positive association with aTB over LTBI (ESAT-6/CFP-10-specific). Additionally, multiparametric FlowSOM analyses revealed an increase in CD27 cell clusters and a decrease in HLA-DR cell clusters within *Mtb*-specific populations after the end of treatment.

**Conclusion:**

Our study independently confirms that CD27^−^, CD38^+^, HLA-DR^+^ and Ki-67^+^ populations on *Mtb*-specific CD4^+^ T-cells are increased during active TB disease. Multiparametric analyses unbiasedly identify clusters based on CD27 or HLA-DR whose abundance can be related to treatment efficacy. Further studies are necessary to pinpoint the convergence between conventional and multiparametric approaches.

## Introduction

To date, tuberculosis (TB) is still a major global health threat, with approximately 10 million new *Mycobacterium tuberculosis* (*Mtb*) infections and 1.5 million deaths reported only in 2020. Moreover, the COVID-19 pandemic has set back several years of progress in TB control, and the target of ending the epidemic by 2030 set by the WHO’s End TB Strategy seems even more implausible now (Global Tuberculosis Report, 2021). From a pathophysiological setting, TB represents a dynamic spectrum from infection to clinical disease (Furin et al., 2019); however, for pragmatic reasons, patients are usually categorized as having either active TB (aTB) or latent TB infection (LTBI). Defining the latent TB condition has been a matter of discussion over the last decades, since it encompasses a wide array of situations, from individuals with quiescent *Mtb* to individuals with controlled bacterial growth for months (Pai et al., 2016). Updated definitions of LTBI added the concept of host immunity to the clinical picture, and now it is considered as “a state of persistent immunoreactivity to *Mtb* antigenic stimulation with no evidence of clinically manifest active TB” (Drain et al., 2018). Around a quarter of the world’s population is estimated to be infected with *Mtb,* from which 5%–10% will develop aTB (Houben and Dodd, 2016). Individuals with LTBI constitute the principal reservoir for the bacilli, which is the reason why accurate diagnosis is essential for halting TB worldwide. Correct characterization of the TB spectrum is, therefore, a priority in the development of novel diagnostic tools.

The immune response to *Mtb* infection is still not fully understood, but CD4^+^ T-cells arguably play a pivotal role (Gallegos et al., 2011; O’Garra et al., 2013). Depletion of this T-cell subset and/or Th1 responses (Filipe-Santos et al., 2006) result in extreme susceptibility to the disease. In fact, immune response to *Mtb* is so connected to T-cell activity that its measurement is the basis for the main culture-free diagnostic assays (Lalvani and Pareek, 2010), such as the Tuberculin Skin Test (TST) and the interferon (IFN)-gamma (γ) release assays (IGRAs). The TST consists in the intradermal injection of a mixture of mycobacterial antigens, the Purified Protein Derivative (PPD), that causes a skin reaction if there is an immunological memory to *Mycobacterium* spp. (Seddon et al., 2016). Despite being widely used, the TST has a rather low specificity, and false positives are common. This is mainly due to the fact that the PPD antigens are not unique to *Mtb* and are detected by T-cells from patients with the Bacille Calmette-Guérin vaccine and/or infected with nontuberculous mycobacteria (Domínguez et al., 2008; Latorre et al., 2010). These limitations were partly overcome thanks to the introduction of the IGRAs. These assays are based on the detection of specific T-cell responses against *Mtb*, represented by the release of IFN-γ after stimulation with *Mtb*-specific antigens (ESAT-6 and CFP-10; Andersen et al., 2000). The IGRAs have demonstrated better performance than the TST for the diagnosis of *Mtb* infection (Metcalfe et al., 2013); nevertheless, both tests fail to discriminate active disease from latent infection and other status associated to higher risks of developing the disease (Goletti et al., 2022). Moreover, neither of the assays can be used to monitor therapy efficacy, which would be of great value to shorten treatment regimen and to accelerate the drug evaluation in the clinical trials. Given the fact that T-cells change their activation status and cytokine expression after *Mtb* challenge, the evaluation of their immune profile is an attractive option for the improvement of TB diagnostics.

The study of immunological biomarkers *via* flow cytometry has already been demonstrated to be a useful tool to evaluate host response in TB infection and aTB disease (Pollock et al., 2013; Coppola et al., 2020). Most studies focus the analysis of these disease markers in specific subsets of cells defined by the expression of pro-inflammatory cytokines following antigen stimulation, linking production of TNF-α (Harari et al., 2011; Halliday et al., 2017) and IFN-γ (Petruccioli et al., 2015; Ahmed et al., 2019) with infection. Among these cells, several surface and intracellular proteins have been described as potential biomarkers for aTB (Walzl et al., 2018). The present research explores the role of four of them; namely, CD27, CD38, HLA-DR and Ki-67. CD27 is a maturation marker and its downregulation has already been associated with active disease and tissue destruction in TB (Portevin et al., 2014; Latorre et al., 2019). CD38 is a transmembrane receptor expressed in many immune cell types (Hartman et al., 2010), more recently linked to TB studies because of its strong expression in activated T-cells. HLA-DR is an MHC cell receptor highly present on APCs (Saraiva et al., 2018), but also with robust expression on activated T-cells (Tippalagama et al., 2021). Ki-67, the only intracellular marker from the group, is a nuclear protein commonly associated with cell proliferation (Soares et al., 2010). The latter three proteins are associated with antigenic stimulation; therefore, their expression is likely to be increased in active forms of the disease, where mycobacterial load is higher (Mpande et al., 2021). In this paper, we aim to study the expression of the CD27, CD38, HLA-DR and Ki-67 markers in *Mtb*-specific CD4^+^ T-cells by performing a side by side comparison of their performance on characterizing active disease and latent infection. Furthermore, we intend to evaluate the capacity of these biomarkers to assess treatment efficacy after its completion. Deeper knowledge on such immune biomarkers could allow the development of new strategies for diagnosis and management of LTBI individuals and aTB patients.

## Materials and methods

### Study population and inclusion criteria

The patients included in this study (*n* = 45) attended one of the following centers in Barcelona, Spain: Hospital Germans Trias i Pujol, Unitat de Tuberculosi Vall d’Hebron-Drassanes, Sant Joan Despí Moisés Broggi, and Hospital Universitari Vall d’Hebrón. They were classified as follows: (i) twenty-three patients (*n* = 23) with pulmonary aTB, microbiologically confirmed with a positive culture and/or PCR, with less than 1 month of anti-TB treatment; (ii) twenty-two individuals (*n* = 22) with LTBI based on positive TST and/or IGRAs and absence of clinical symptoms and radiological signs, detected by contact-tracing or screening studies, and with less than 1 month of chemoprophylaxis; and (iii) nine (*n* = 9) former aTB patients from group (i) after successfully completing the standard treatment regimen with anti-TB drugs and being considered as cured (eTrt). Overall, 37% were women, and the mean age (years) ± standard deviation (SD) was 42.7 ± 13.5. Clinical and study information is detailed in Table 1.

**Table 1:** Demographic and clinical characteristics of the participants in each study group.

Variables	aTB	LTBI	eTrt
Participants, *n*	23	22	9
Mean age^1^, years ± SD	43.91 ± 15.47	42.59 ± 12.17	50 ± 14.02
Male gender, *n*(%)	18 (78.3)	16 (72.7)	9 (100)
Disease form, N(%)			
Pulmonary	22 (95.7)	–	9 (100)
Pleural	1 (4.3)	–	–
Reported LTBI enrolment, N(%)			
Contact-tracing	–	14 (63.6)	
LTBI screening	–	7 (31.8)	
Not reported	–	1 (4.5)	
Chemoprofilaxis, N(%)			
Before starting chemoprophylaxis	-	1 (4.5)	-
After starting chemoprophylaxis (<1 month)	22 (95.7)	21 (95.5)	9 (100)
Not prescribed	-	-	-
Mean time of chemoprophylaxis^2^, days ± SD	-	20.90 ± 6.32	-
Regimen 3RH	-	19 (86.4)	-
Regimen 6H	-	3 (13.6)	
Anti-TB treatment, N(%)			
Before starting treatment^3^	2 (8.7)	–	–
After starting treatment (<1 month)	21 (91.3)	–	–
Ended treatment^4^	-		9 (100)
Not prescribed	-	22 (100)	–
Mean time of treatment^2^, days ± SD	12.95 ± 9.94	–	229.55 ± 91.48
Regimen 2HRZE/4RH	21 (91.3)	–	8 (88.9)
Others^5^	2 (8.7)	–	1 (11.1)
Comorbidities, N(%)			
Other respiratory disorders (asthma, PCD, COPD)	2 (8.7)	2 (9.1)	2 (22.2)
Neoplasies (lung, prostate)	2 (8.7)	–	1 (11.1)
Autoimmune diseases (diabetes, psoriasis, Löfgren Syndrome)	2 (8.7)	2 (9.1)	–
Cardiovascular diseases (AHT, cardiomyopathy)	1 (4.3)	–	1 (11.1)
Hepatitis C	1 (4.3)	2 (9.1)	–
Bacille Calmette-Guérin vaccine, N(%)			
BCG-vaccinated	12 (52.2)	12 (54.5)	3 (33.3)
Not BCG-vaccinated	9 (39.1)	10 (45.5)	5 (55.5)
Not reported	2 (8.7)	–	1 (11.1)
Other information, N(%)			
Reported smokers	9 (39.1)	5 (22.7)	4 (44.44)
Reported drug abuse	5 (21.7)	0 (0)	0 (0)

aTB, active tuberculosis; LTBI, latent tuberculosis infection; eTrt, aTB patients after anti-TB treatment; SD, standard deviation; PCD, primary ciliary dyskinesia; COPD, chronic obstructive pulmonary disease; AHT, arterial hypertension; SEB, staphylococcal enterotoxin B.

1 Patients in the study are aged 20–73.

2 Range of time between starting chemoprophylaxis and anti-TB treatment and sample collection was from 0 to 30 days in all participants in both groups.

3 Both patients were prescribed with RIMSTAR on the day of sample collection.

4 One of the participants was recruited after 5 months of anti-TB treatment, six of the participants between 6 and 7.5 months and one of the participants after 16 months.

5 One patient was prescribed with HRZ regimen, and one patient was prescribed with Lzd/Mfx/Cfz/Z/E regimen.

### PBMCs isolation, preservation, and thawing

Approximately 16 ml of whole blood were collected from each patient in cell preparation tubes (CPT tubes; BD Biosciences, San Jose, CA, United States) containing Sodium Citrate and Ficoll for peripheral blood mononuclear cells (PBMCs) isolation. Briefly, following a centrifugation of 1,600 g for 30 min, PBMCs were harvested from the interface and washed with RPMI medium (ThermoFisher, Waltham, MA, United States) supplemented with 10% FBS (BioWest, Miami, Fla., United States). Cells were counted with Trypan Blue staining and were resuspended in heat-inactivated FBS supplemented with 10% Dimethyl Sulfoxide (DMSO) for cryopreservation in liquid nitrogen (Cossarizza et al., 2019). To perform our immunological studies, frozen PBMCs were thawed and incubated for 2 h at 37°C with 5% CO2 in AIM-V medium (Thermo Fisher) with benzonase (Sigma, St. Louis, MO, United States; final concentration 10 U/ml) to avoid cell clumping.

### PBMCs stimulation with mycobacterial antigen

Samples included in the study were stimulated for 16 h separately with two mycobacterial antigen mixes: (i) recombinant proteins ESAT-6/CFP-10 (Lionex Diagnostics and Therapeutics, Braunschweig, Germany; final concentration 2 μg/ml each) and (ii) PPD (AJVaccines, Copenhagen, Denmark; final concentration 10 μg/ml). A positive control consisting of staphylococcal enterotoxin B (SEB, Sigma, final concentration of 2 μg/ml) and a negative control without stimulation were also included for each sample. One million (10^6^) PBMCs were used in each stimulation condition, adding as co-stimulators anti-CD28 and anti-CD49d monoclonal antibodies (BD Bioscience; final concentration 1 μg/ml each). After a 2 h incubation at 37°C in a 5% CO_2_ atmosphere, Brefeldin A (BFA; Sigma; final concentration 3 μg/ml) and Monensin (BioLegend, San Diego, USA, final concentration 1X) were added to inhibit intracellular vesicular transport. Cells were then incubated overnight before starting the staining procedure.

### Extracellular and intracellular staining and acquisition in flow cytometer

After stimulation, PBMCs were labeled with viability marker LIVE/DEAD Near-IR fluorescent reactive dye (Thermo Fisher) for 30 min and subsequently stained for 20 min with the following surface markers: anti-CD3-PerCP (BioLegend), anti-CD4-BV786, anti-CD8-BV510, anti-CD27-BV605, anti-CD38-PE, and anti-HLA-DR-BV421 (BD Bioscience). Cells were then fixed and permeabilized with the Foxp3 Transcription Factor Staining Buffer Set (Thermo Fisher) and stained for 30 min with intracellular markers: anti-IFNγ-APC, anti-TNFα-PE-Cy7 and anti-Ki-67-FITC (BD Bioscience). The complete list of antibodies, conjugated proteins and dilutions can be found in Supplementary Table 1. All incubation processes were performed at room temperature in darkness. Fluorescence Minus One (FMO) controls of the four analyzed cell markers (CD27, CD38, HLA-DR, and Ki-67) were included in each run to accurately distinguish negative from positive populations. Samples were resuspended in 100 μl of PBS-0.1%BSA (Bovine Serum Albumin, Sigma Aldrich) and acquired in a BD LSRFortessa flow cytometer (BD Bioscience) using FACSDiva software (BD Biosciences) with compensated parameters.

### Flow cytometry data analysis

For gate-driven analyses, flow cytometry data was analyzed using FlowJo™ (Tree Star, Ashland, OR, United States) and plotted with GraphPad Prism (GraphPad Software, La Jolla, CA). Lymphocytes were gated based on their size (FSC) and complexity (SSC). After exclusion of doublet events, CD4^+^ T cells were selected from CD3^+^ alive events. TNF-α^+^ and/or IFN-γ^+^ populations were selected for the study of *Mtb-*specific responses. To avoid inadequate assessment of population percentages, *Mtb-*specific populations under 100 events were not considered valid for analysis (this meant that out of 54 samples, six PPD-stimulated and eight ESAT-6/CFP-10-stimulated were excluded). CD27 Median Fluorescence Intensity (MFI) ratio


$$\left[\frac{\mathrm{CD}27\;\mathrm{MFI}\;\;\mathrm{on}\;\mathrm{total}\;\mathrm{CD}4^{+}\;T-\mathrm{cells}\;}{\mathrm{CD}27\;\mathrm{MFI}\;\mathrm{on}\;Mtb-\mathrm{specific}\;\mathrm{CD}4^{+}\;T-\mathrm{cells}}\right]$$

and ΔHLA-DR MFI


$$\left(\begin{array}{l} \mathrm{HLADR}\;\mathrm{MFI}\;\mathrm{on}\;{\mathrm{IFNy}}^{+}\\ {\mathrm{TNF}}^{+}\mathrm{CD}3^{+}\mathrm{cells} \end{array}\right)-\left(\begin{array}{l} \mathrm{HLADR}\;\mathrm{MFI}\;\mathrm{on}\;\\ \mathrm{total}\;\mathrm{CD}3^{+}\mathrm{cells} \end{array}\right)\;$$

were calculated according to published reports led by Portevin et al. (2014) and Mpande et al. (2021), respectively.

For multiparametric analyses, dimensional reduction and clustering were done using OMIQ data analysis software (OMIQ, Inc. Santa Clara, CA) after preliminary cleaning of data with FlowJo of aggregates, dead cells and debris. All CD3 positive events from all samples were selected for subsequent analysis on the OMIQ platform. flowCut algorithm was run to check and exclude for any aberrant regions of all files analyzed. Subsequently, a UMAP analysis was performed to visualize the different CD3 subsets in groups. FlowSOM was run to cluster the data using metaclustering with *k* = 50. After the FlowSOM analysis, the metaclusters were grouped into commonly recognized biological populations. All clusters were plotted on traditional dot plots for phenotype confirmation as for the standard manual gating analysis.

### Statistics

Results comparing variables between LTBI and aTB participants were analyzed using the two-tailed Mann–Whitney test for unpaired comparisons. Paired data from patients followed over time was analyzed using the Wilcoxon-matched pairs test. Comparisons between before and after treatment groups where data was partially paired (combining paired and unpaired observations) were analyzed using a mixed statistical model controlling repeated measures on logit-transformed data. Correlation between variables was calculated using the two-tailed non-parametric Spearman test. Diagnostic accuracy was evaluated *via* the Receiver operating characteristic (ROC) and Area Under the Curve (AUC) analyses. Differences were regarded as statistically significant when the value of *p* or the False Discovery Rate (FDR) value were below 0.05.

## Results

### Expression of CD27, CD38, HLA-DR and Ki-67 markers on *Mtb*-specific CD4^+^ T-cells differs in LTBI individuals and anti-TB treated patients compared to aTB patients

To specifically study the T-cell response against *Mtb*, TNF-α and/or IFN-γ cytokines were analyzed on CD4^+^ T-cells after stimulation with ESAT-6/CFP-10 or PPD antigens (complete gating strategy can be found in Supplementary Figure 1). From this *Mtb-*specific population, the percentage of CD27^−^, CD38^+^, HLA-DR^+^ and Ki-67^+^ populations were compared between LTBI individuals and aTB patients at the beginning and end of the treatment. To control that the length of the treatment within the 1-month range of the aTB group did not interfere with the marker’s expression, we performed a Spearman Correlation Test that showed no correlation between the days of treatment in month 1 and our variables (Supplementary Figure 2).

**Figure 1:**
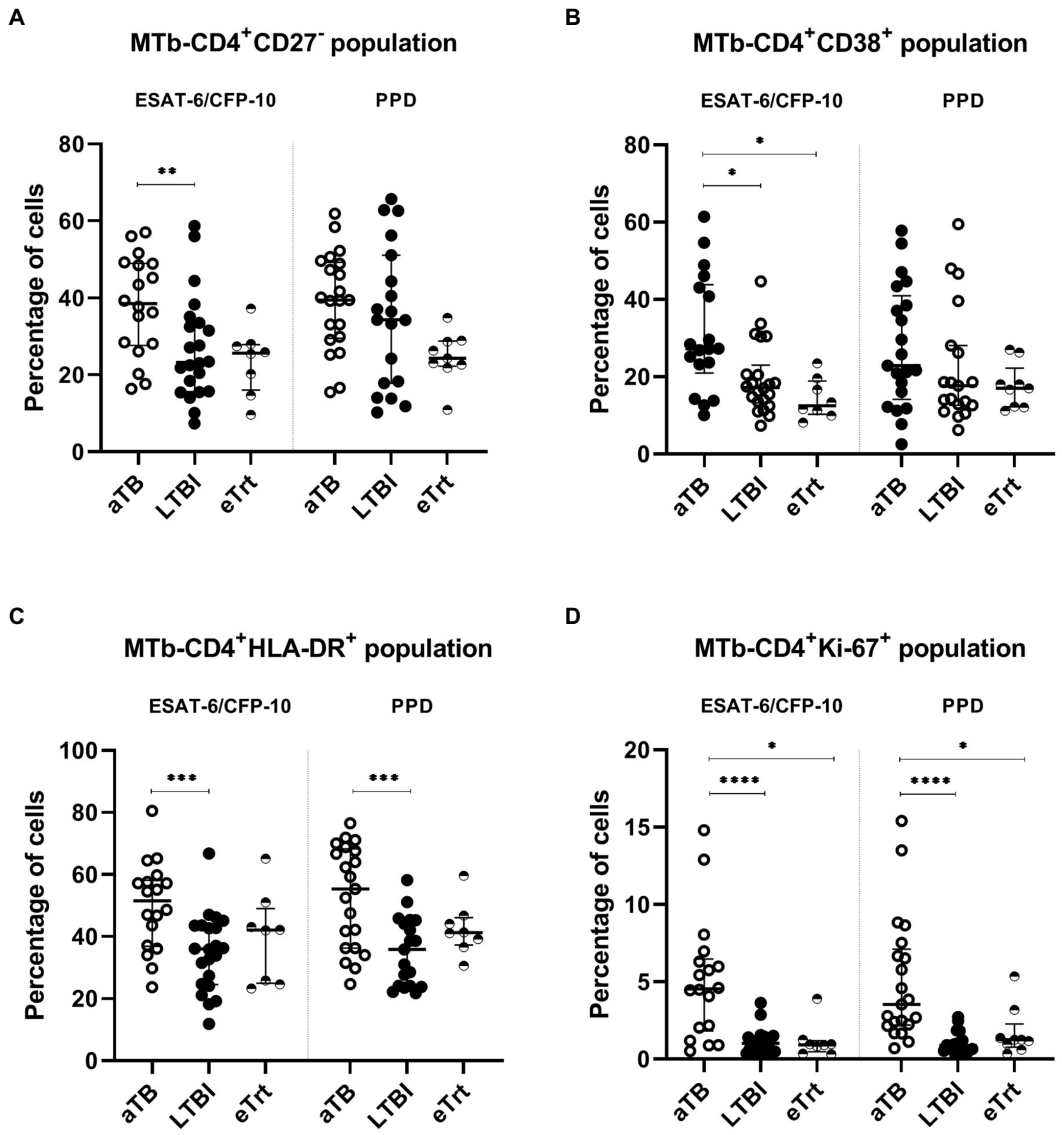
CD27^−^, CD38^+^, HLA-DR^+^, and Ki67^+^ phenotype from Mtb-specific CD4^+^ T-cells of each patient group. Percentage of CD27^−^**(A)**, CD38^+^ **(B)**, HLA-DR^+^ **(C)**, and Ki67^+^ **(D)** within TNF-α^+^ and/or IFN-γ^+^ CD4^+^ T-cells after stimulation with ESAT-6/CFP-10 or PPD (left and right half of graph, respectively) in patients with active TB in the beginning and end of treatment, as well as LTBI individuals. Data plotted with median and interquartile range. Differences between aTB and LTBI conditions were calculated using the two-tailed Mann–Whitney *U*-test. Differences between aTB and eTrt groups were calculated using a mixed statistical model controlling repeated measures on logit-transformed data. ^*^p < 0.05, ^**^p < 0.01, ^***^p < 0.001, ^****^p < 0.0001. No indication of *p* value implies non significance. aTB, active TB; LTBI, latent tuberculosis infection; eTrt, aTB patients after anti-TB treatment.

As shown in Figures 1A–D, the percentage of all populations studied (CD27^−^, CD38^+^, HLA-DR^+^ and Ki-67^+^) within *Mtb-*specific CD4^+^ T-cells was increased in aTB compared to LTBI in response to ESAT-6/CFP-10 recombinant proteins (*p* = 0.0092 for CD27^−^, *p* = 0.0162 for CD38^+^, *p* = 0.001 for HLA-DR^+^ and *p* < 0.0001 for Ki-67^+^). Additionally, the HLA-DR^+^ and Ki-67^+^ populations were also associated with aTB patients after stimulation with PPD (*p* = 0.0008 and *p* < 0.0001, respectively). The trends observed in individuals who finished the anti-TB regimen were comparable to those in LTBI individuals. As seen again in Figure 1, following treatment, a decrease in the percentages of all the phenotypes was observed, with significance for the CD38 (*p* = 0.0384 for ESAT-6/CFP-10) and Ki-67 (*p* = 0.0293 for ESAT-6/CFP-10, *p* = 0.022 for PPD) markers. Reduction of CD27^−^ and HLA-DR^+^ populations in activated CD4^+^ T-cells was statistically significant only when performing a Mann–Whitney unpaired analysis excluding the matched samples (for CD27^−^, *p* = 0.0016 and *p* = 0.0001 and for HLA-DR^+^, *p* = 0.0482 and *p* = 0.0649 after stimulation with ESAT-6/CFP-10 and PPD, respectively). No differences were detected on any of the markers’ expression regarding BCG-status after PPD stimulation (Supplementary Figure 3). When analyzing only the patients monitored at the beginning and at the end of treatment, inter-individual variation of the marker’s expression was observed (Supplementary Figure 4).

### CD27 MFI ratio provides a useful tool to characterize aTB

**Figure 2:**
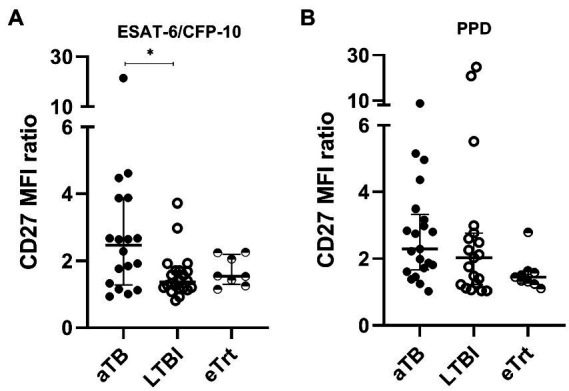
CD27 MFI ratio is increased in aTB patients over LTBI individuals (ESAT-6/CFP-10-specific) and patients after anti-TB treatment (PPD-specific). A ratio of CD27 MFI was calculated as suggested by Portevin et al. The numbers result from the division of the MFI of CD27 in CD4^+^ T-cells over MFI of CD27 in TNF-α^+^ and/or IFN-γ^+^ CD4^+^ T-cells. **(A)** CD27 MFI ratio after ESAT-6/CFP-10 or **(B)** PPD stimulation in patients with active TB in the beginning and end of treatment, as well as LTBI individuals. Data plotted with median and interquartile range. Differences between aTB and LTBI conditions were calculated using the two-tailed Mann–Whitney *U*-test. Differences between aTB and eTrt groups were calculated using a mixed statistical model controlling repeated measures on logit-transformed data. ^*^p < 0.05. aTB, active TB; LTBI, latent tuberculosis infection; eTrt, aTB patients after anti-TB treatment.

We also evaluated an alternative approach based on the ratio between the CD27 MFI in total CD4^+^ T-cells and the MFI of CD27 in *Mtb-*specific CD4^+^ T-cells. An increase of this ratio is a direct consequence of a decrease of the CD27^+^ *Mtb-*specific CD4^+^ T-cells phenotype associated with active disease. Overall, our data shows a reverse trend of the CD27 MFI ratio with aTB to that observed in the percentage of CD27^−^ cells among the *Mtb*-specific CD4^+^ cells. As seen in Figure 2A, the ratio of CD27 MFI was increased in aTB patients compared to LTBI individuals after ESAT-6/CFP-10 stimulation of T-cells (*p* = 0.0162) (Figure 2B). A difference was found when comparing aTB and eTrt groups after PPD stimulation when performing a Mann–Whitney unpaired analysis excluding the matched samples (*p* = 0.0044). In order to demonstrate the association between the proportion of CD27^−^ *Mtb*-specific CD4^+^ T-cells and the CD27 MFI ratio, we performed a Spearman’s correlation test (Supplementary Figure 5). A positive interdependence between the two variables was detected in T-cells responding to both ESAT-6/CFP-10 and PPD, supported by a strong correlation coefficient (for ESAT-6/CFP-10: Spearman’s rho = 0.8143, *p* < 0.0001; for PPD: Spearman’s rho = 0.8766, *p* < 0.0001).

Additionally, we calculated the ΔHLA-DR MFI (difference in MFI of HLA-DR between total T-cells and *Mtb*-specific T-cells) to assess its performance in discriminating the three study groups. An increase of this value was observed on aTB patients over LTBI individuals after stimulation with ESAT-6/CFP-10 (*p* = 0.0341; Supplementary Figure 6). MFI ratios of CD38 and Ki-67 were also analyzed but showed no difference between study groups (data not shown).

### Ki-67^+^ and HLA-DR^+^ populations yield the highest discriminative performance

ROC curve analyses were performed for both ESAT-6/CFP-10 and PPD stimulations for each marker in order to explore their diagnostic accuracy of aTB patients over the LTBI condition (Table 2). As expected, highest AUC values (95% confidence interval, CI) corresponded to the marker which provided the highest significant discrimination between aTB and LTBI, Ki-67 [for ESAT-6/CFP-10, AUC 0.87 (0.75–0.99), *p* < 0.0001; for PPD, AUC 0.92 (0.83–1.00), *p* < 0.0001]. Albeit lower, good AUC values were also obtained for HLA-DR marker with strong significance [for ESAT-6/CFP-10, AUC 0.80 (0.65-0.94), *p* = 0.0014; for PPD, AUC 0.80 (0.67–0.94), *p* = 0.0007]. All ROC Curves, as well as sensitivity and specificity cut-off values, can be found in Supplementary Figure 7.

**Table 2:** ROC curve analysis of each marker used in the study.

Marker	AUC (95% CI), *p*	
	ESAT-6/CFP-10	PPD
CD27^−^ Mtb-specific CD4^+^ T-cells	0.7386 (0.5805-0.8968), 0.0120	0.5714 (0.3841-0.7588), 0.4402
CD38^+^ Mtb-specific CD4^+^ T-cells	0.7222 (0.5563-0.8881), 0.0167	0.6078 (0.4268-0.7888), 0.2442
HLA-DR^+^ Mtb-specific CD4^+^ T-cells	0.7967 (0.6514-0.9421), 0.0140	0.8020 (0.6679-0.9361), 0.0011
Ki67^+^ Mtb-specific CD4^+^ T-cells	0.8699 (0.7533-0.9866), <0.0001	0.9198 (0.8380-1.0000), <0.0001
CD27 ratio MFI	0.7222 (0.5473-0.8972), 0.0167	0.6015 (0.4199-0.7831), 0.2727

AUC, area under the curve; CI, confidence interval.

### Multiparametric analyses reveal the presence of cell subsets with significant differences in abundance in aTB patients before and after treatment

**Figure 3:**
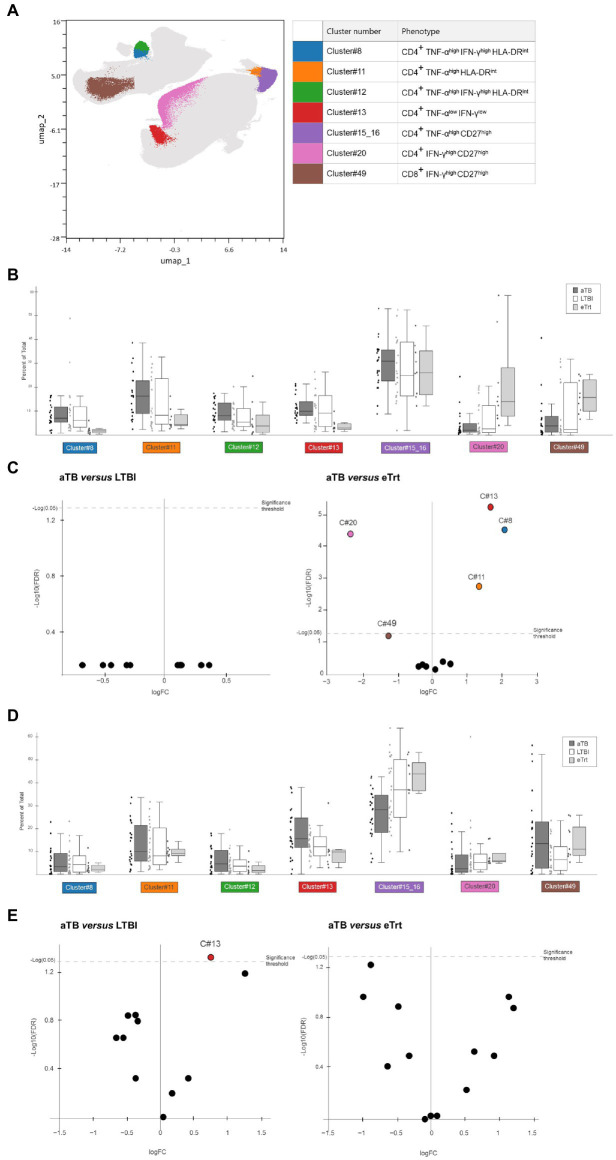
Results from multiparametric analyses of the samples. **(A)** UMAP based on the expression of CD4, CD8, TNF-α, IFN-γ, CD27, CD38, HLA-DR and Ki-67 markers in CD3^+^ cells from our dataset containing LTBI individuals and aTB patients before and after treatment, after stimulation with ESAT-6/CFP-10 or PPD. Colored clusters indicate populations with difference in abundance depending on disease status, obtained with FlowSOM and analyzed within TNF-α^+^ and/or IFN-γ^+^ subsets. In background, all 50 cell clusters defined. **(B,D)** Box and dot plots showing percentage (Y axis) of each cluster of interest in each respective group [**(B)** for samples after PPD stimulation, **(D)** for samples after ESAT-6/CFP-10 stimulation. Data plotted with median and interquartile range. **(C,E)** Volcano plot displaying logarithm scale of fold-change of percentage ratio of sample from aTB patients over LTBI individuals (left) and patients who completed treatment (right; **C**) for samples after PPD stimulation, **(E)** for samples after ESAT-6/CFP-10 stimulation]. Positive values (logFC>1) show clusters whose proportion is increased in aTB, while negative values (logFC<−1) show clusters whose proportion is decreased in aTB. Significant samples are represented above the threshold (*q* < 0.05). Volcano plots were automatically produced by the OMIQ software. aTB, active TB; LTBI, latent tuberculosis infection; eTrt, aTB patients after anti-TB treatment, UMAP: uniform manifold approximation; FDR: false discovery rate.

To avoid the analytical bottleneck produced by manual gating, we aimed to explore the same cytometry data in an unbiased manner using a multiparametric analysis. This approach offered the possibility of defining complex cell phenotypes that cannot be revealed using traditional biaxial data presentation. We imported the data on CD3^+^ populations after ESAT-6/CFP-10 or PPD stimulations into OMIQ and analyzed the data using Uniform Manifold Approximation and Projection for Dimension Reduction (UMAP) and FlowSOM for clustering populations. In total, 50 different cell clusters were defined, being 11 of them positive for TNF-α and/or IFN-γ. Within this subset, at least seven clusters displayed significant changes in expression between the three study groups (Figure 3A). Some of these clusters presented similar features and could broadly be classified into a “maturation phenotype” [CD4^+^ CD27^+^ cells with expression of TNF-α (cluster #15_16) or IFN-γ (cluster #20)] or an “activation phenotype” [CD4^+^ HLA-DR^int^ cells with expression of TNF-α (cluster #11) or both TNF-α and IFN-γ (clusters #8, #12)]. One of the clusters did not show our markers of interest and just presented low expression of cytokines TNF-α and IFN-γ (cluster #13).

Figures 3B,C show the results obtained for samples stimulated with PPD. As it can be seen, the percentage of population of each cluster was compared between the aTB, LTBI and eTrt groups (Figure 3B). No differences were found between the aTB and LTBI groups for any cluster. However, as evidenced by median log_2_ fold-changes [log_2_FC(aTB/eTrt) > 1 or < −1 when increased or decreased in aTB samples, respectively] and confirmed by FDR analyses (*q* < 0.05), clusters #8, #11, and #13 were significantly increased in aTB patients, whereas cluster #20 was significantly increased in patients who completed treatment (Figure 3C). An additional cluster of CD8^+^IFN-γ^+^CD27^+^ cells (cluster #49) was also identified with a positive association with cured patients (*q* = 0.059). The same analysis was performed for samples stimulated with ESAT-6/CFP-10 antigens, showing that cluster #13 was more prominent in active TB patients than in LTBI individuals (Figures 3D,E). Together, data from multiparametric analyses show a potential role of CD27 and HLA-DR clusters in differentiating aTB patients before and after treatment, and warrant more research using unbiased analyses.

## Discussion

Recently, the field of TB diagnosis and management has seen a rise in the development of tools based on the evaluation of the host immune features. In this study, we have analyzed the expression profile of different biomarkers in specific T-cell subsets stimulated with *Mtb* antigens. We have reported modulations on the expression of the four cell markers in *Mtb-*specific CD4^+^ T-cells, dependent on the presence of clinical disease or infection alone, and that also reflect therapy efficacy. Additionally, we have provided data from unbiased multiparametric analyses showing the presence of several cell clusters with potential to characterize different disease status.

The results of our conventional analyses show the performance of CD27, CD38, HLA-DR and Ki-67 to discriminate active disease from latent infection. Regarding CD27 receptor, our data suggests that its expression on *Mtb*-specific CD4^+^ T-cells is down-regulated during active disease. These results are in line with most of the published data; in fact, CD27 is, out of the four proteins of our study, the one with more extensive literature, both in mice (Lyadova et al., 2004) and human (Petruccioli et al., 2016; Riou et al., 2017; Acharya et al., 2021; Xu et al., 2021). CD27 is a maturation marker expressed by lymphocytes associated with lack of responsiveness toward different antigens (Schiött et al., 2004), and downregulated in effector differentiated T-cells (Nikitina et al., 2012). Therefore, it can be expected that CD27^−^ populations are increased in aTB patients. The rationale behind the discriminative power of the other three markers is simple: they are related to the activation and/or enhancement of immune responses, which commonly follow pathogenic infections. Our data gathered on these also confirms what has been published on the topic: CD38 expression on activated CD4^+^ T-cells was increased in aTB patients after ESAT-6/CFP-10 stimulation (Luo et al., 2021), but HLA-DR^+^ and Ki-67^+^ populations showed the most significant association with active disease in all conditions. These findings are consistent with most of the previous research in the field (Adekambi et al., 2015; Silveira-Mattos et al., 2020). AUC values obtained for the discriminating performance of these two proteins, especially Ki-67, greatly surpass those from CD27, indicating that activation markers might have more potential than maturation markers on conventional analyses.

In this paper we also evaluated the ratio of CD27 MFI as an alternative way to measure the expression of the biomarkers. This method, proposed by Portevin et al. (2014), added a technique to discriminate aTB from LTBI similar to measuring CD27^−^ populations but allowing normalization of results, thus preventing discrepancies raised by positive or negative gating. Here, we show that CD27 MFI quantification mirrors the data obtained by the analysis of percentage of CD27^−^. This was further supported using Spearman’s Correlation test, which shows positive interdependence between both variables. We also performed an analysis on the ΔHLA-DR median fluorescence intensity biomarker following recent work in South Africa (Mpande et al., 2021), and it provided an even additional technique for distinguishing latent from active infection. These findings warrant further studies on this approach, either for validation with the same biomarkers or implementation on other ones.

Another objective of this research was to study the efficacy of the markers in evaluating anti-TB treatment efficacy. The hypothesis followed was that participants with a successful response to treatment developed similar immunophenotypes to individuals without clinical manifestations. Despite the fact that data on treatment monitoring is sparse, some studies have shown that T cell activation markers are reduced after treatment (Vickers et al., 2020), and that the same markers can be used to identify both LTBI and cured individuals (Ahmed et al., 2018) or predict relapse (Goletti et al., 2018). In our study, a small cohort of patients who completed treatment showed a significant decrease on the expression CD38^+^, and Ki-67^+^ phenotypes, compared to those at the beginning of treatment. Despite the size difference between the groups, our results agree with what has been published on the matter (Hiza et al., 2022). We did not find statistical significance differences in our mixed model for the HLA-DR and CD27 expression after anti-TB treatment; however, this can be explained by the relatively small sample size of the group of patients who completed therapy. This limitation was also present when analyzing each patient monitored individually. We expect to carry out future studies with the necessary population size to state robust conclusions.

The unbiased, multiparametric analysis provides an additional approach that is in line with literature on the evaluation of therapy efficacy *via* manually gated analyses. The abundance of clusters expressing CD27 was increased after treatment, as commonly do the CD27^+^ populations in conventional analysis; and so occurred with HLA-DR^+^ clusters and populations in the case of patients with aTB. It should be noted that Ki-67, the most discriminative marker, was not defined in any of our 50 clusters. However, given that most clustering techniques are more accurate when the number of data points is high, it might be possible that the low cell count in Ki67^+^ populations posed a difficulty in the identification of such subsets. It seems that the multiparametric analyses might open the door for investigating unique combinations of biomarkers, but much further work is required to establish and standardize this technique.

There are limitations in this study that should be addressed. First, the strategy chosen for the definition of *Mtb-*specific cells relies on the measurement of IFN-γ and TNF-α, since we already showed that CD27 profile was similar in populations positive for one or both cytokines (Latorre et al., 2019). However, not all *Mtb-*specific CD4^+^ T-cells express these cytokines (Morgan et al., 2021). Other approaches for the characterization of this subset (e.g., measurement of other cytokines or multimeric tetramer staining) should be considered. And second, the immune status of each patient depends on multiple parameters, involving not only host and pathogen genetics but also the age of the host (Veneri et al., 2009), the length and type of antibiotic treatment and the recency of infection (Amiano et al., 2020), among others. Therefore, it is necessary to take into account the inherent heterogeneity of the patients when translating potential biomarkers into clinical applications. The use of healthy controls in future work could help establish the baseline expression of these markers and highlight their diagnostic potential. Another issue to address in future studies is the miniaturization of the assay for its implementation as point of care testing. Although flow cytometry studies are generally unwieldy, they can be simplified as evidenced by recent work on HLA-DR-based (Musvosvi et al., 2018) and CD38-based (Hiza et al., 2021) rapid assays. Reducing diagnostic waiting times, test difficulty and costs, among others, is of utmost importance to make the test deployable in areas with high LTBI burden.

In conclusion, our findings on maturation and activation markers CD27, CD38, HLA-DR and Ki-67 on *Mtb*-specific CD4^+^ T-cells confirm their promising role as potential TB biomarkers for the characterization of LTBI and aTB. Moreover, we provide data showing that after finishing therapy, the immune profile of these markers resembles that of LTBI individuals. It is crucial to evaluate these results in larger cohorts of patients to validate its performance and to study the possibilities of implementation with other clinical signs and microbiology tests. We also show how multiparametric profiling of *Mtb*-specific cell subsets can lead to the discovery of unique cell clusters with different expression across study groups of aTB, LTBI and cured patients. More research on the topic is necessary in order to pinpoint the link between the immune phenotype and the different stages of the disease.

## Data availability statement

The raw data supporting the conclusions of this article will be made available by the authors, without undue reservation.

## Ethics statement

The studies involving human participants were reviewed and approved by the Hospital Germans Trias i Pujol (reference number PI-19-149). The patients/participants provided their written informed consent to participate in this study.

## Author contributions

IL and RV-H designed the study. IL, MF, RV-H, and SD-F designed the experiments. GT, GS, IR, IL, RV-H, and SD-F performed the experiments. AS-M, CP, MG, MJ-F, JS, JA, and ZS contributed with resources. IL, MF, and SD-F analyzed the data. IL and JD supervised the study. SD-F wrote the paper. All authors contributed to the article and approved the submitted version.

## Funding

This research was supported by a grant from the Instituto de Salud Carlos III (PI18/00411, PI19/01408 and CP20/00070), integrated in the Plan Nacional de I + D + I and cofunded by the ISCIII Subdirección General de Evaluación and the Fondo Europeo de Desarrollo Regional (FEDER); a grant from the Sociedad Española de Neumología y Cirugía Torácica (project 25/2016; SEPAR; Barcelona, Spain); and from the European Union’s Horizon 2020 Research and Innovation Programme under the Marie Skłodowska-Curie grant agreement no. 823854 (INNOVA4TB). JD and IL are researchers from the Miguel Servet programme. AS-M is supported by a Juan Rodés (JR18/00022) postdoctoral fellowship from ISCIII.

## Conflict of interest

The authors declare that the research was conducted in the absence of any commercial or financial relationships that could be construed as a potential conflict of interest.

## Publisher’s note

All claims expressed in this article are solely those of the authors and do not necessarily represent those of their affiliated organizations, or those of the publisher, the editors and the reviewers. Any product that may be evaluated in this article, or claim that may be made by its manufacturer, is not guaranteed or endorsed by the publisher.

## Supplementary material

Supplementary Figure 1 Gating strategy used for CD4^+^ T-cells cytokine secretion and markers analysis. **(A)** After rough selection of lymphocytes, aggregated cells were taken off by gating on the diagonal that appears with Forward-Scatter (height; FSC-H) vs. Forward-Scatter (area; FSC-A) dot plot. CD4^+^ T-cells were gated from a parent population of alive CD3^+^ T-cells. **(B)** *Mtb*-specific population were selected from parent population CD4^+^ T-cells and gated based on the expression of TNF-α^+^ and/or IFN-γ^+^, from which CD27/CD38/HLA-DR/Ki67 expression was studied. The panels are representative of populations found without stimulation (left) and after PPD (middle) and ESAT-6/CFP-10 (right) stimulation. (**C**) Panels showing representative examples of cell distribution according to each marker of the study (sample shown is from an LTBI individual after PPD stimulation). The gates were decided according to FMO (Fluorescence Minus One) controls.

Supplementary Figure 2 Expression of markers in aTB patients according to the day of treatment within the first month. Correlation between the percentage of CD27^−^, CD38^+^, HLA-DR^+^ and Ki-67^+^ within TNF-α^+^ and/or IFN-γ^+^ CD4^+^ T-cells and the days after the start of the treatment during the first month in patients from the aTB group.

Supplementary Figure 3 BCG vaccination had no effect in the outcome measures of the analysis in PPD-stimulated samples. Percentage of CD27^−^, CD38^+^, HLA-DR^+^, and Ki67^+^ within TNF-α^+^ and/or IFN-γ^+^ CD4^+^ T-cells after stimulation with PPD. aTB patients (up) and LTBI individuals (down) are separated by their vaccination status of BCG. Differences between conditions were calculated using the Mann–Whitney U test. No indication of *p* value implies no significance.

Supplementary Figure 4 Changes in CD27^−^, CD38^+^, HLA-DR^+^, and Ki67^+^ phenotypes of each monitored individual before and after treatment. Percentage of CD27^−^, CD38^+^, HLA-DR^+^, and Ki67^+^ within TNF-α^+^ and/or IFN-γ^+^ CD4^+^ T-cells after stimulation with ESAT-6/CFP-10 (left) or PPD (right) exclusively for the aTB patients that were monitored before (≤1 month) and after (≥6 months) anti-TB therapy. Because of exclusion from one of the points from both ends due to analysis restrictions, only patients with both samples positive for the stimulants are shown. Differences between conditions were calculated using the Wilcoxon signed rank paired test. **p* < 0.05. No indication of *p*  value implies non significance.

Supplementary Figure 5 Strong correlation between the percentage of CD27^−^ in TNF-α^+^ and/or IFN-γ^+^ CD4^+^ T-cells and the CD27 MFI ratio after ESAT-6/CFP-10 (left) and PPD (right) stimulation. Correlation was calculated using the two-tailed non-parametric Spearman test. **p* < 0.05, ***p* < 0.01, *****p* < 0.0001. aTB, active TB; LTBI, latent tuberculosis infection; eTrt, aTB patients after anti-TB treatment.

Supplementary Figure 6 ΔHLA-DR MFI of each respective group. ΔHLA-DR median fluorescent intensity (MFI) was calculated as suggested by Mpande et al. The numbers result from subtracting the HLA-DR MFI on IFN-γ+ and/or TNF+ CD3 + cells to the HLA-DR MFI on total CD3+ cells. **(A)** ΔHLA-DR MFI after ESAT-6/CFP10 or **(B)** PPD stimulation in patients with active TB in the beginning and end of treatment as well as LTBI individuals. Data plotted with median and interquartile range. Differences between aTB and LTBI conditions were calculated using the two-tailed Mann–Whitney  U-test. Differences between aTB and eTrt groups were calculated using a mixed statistical model controlling repeated measures on logit-transformed data. **p* < 0.05.

Supplementary Figure 7 Area under the receiver operating characteristic curve (AUROC) showing performance of CFP-10/ESAT-6–specific (left) and PPD-specific (right) TNF-α+ and/or IFN-γ+ CD4+ T-cells to discriminate between aTB and LTBI individuals for every marker studied in the paper. Se, sensitivity; Sp, specificity.

## References

[ref1] Acharya M. P. Pradeep S. P. Murthy V. S. Chikkannaiah P. Kambar V. Narayanashetty S.. (2021). CD38+CD27-TNF-α+on Mtb-specific CD4+T cells is a robust biomarker for tuberculosis diagnosis. Clin. Infect. Dis. 73, 793–801. doi: 10.1093/cid/ciab144, PMID: 34492697

[ref2] Adekambi T. Ibegbu C. C. Cagle S. Kalokhe A. S. Wang Y. F. Hu Y.. (2015). Biomarkers on patient T cells diagnose active tuberculosis and monitor treatment response. J. Clin. Invest. 125:3723. doi: 10.1172/JCI77990, PMID: 26325038 PMC4588276

[ref3] Ahmed M. I. M. Ntinginya N. E. Kibiki G. Mtafya B. A. Semvua H. Mpagama S.. (2018). Phenotypic changes on *mycobacterium tuberculosis*-specific CD4 T cells as surrogate markers for tuberculosis treatment efficacy. Front. Immunol. 9:2247. doi: 10.3389/fimmu.2018.02247, PMID: 30323818 PMC6172348

[ref4] Ahmed M. I. M. Ziegler C. Held K. Dubinski I. Ley-Zaporozhan J. Geldmacher C.. (2019). The TAM-TB assay-A promising TB immune-diagnostic test with a potential for treatment monitoring. Front. Pediatr. 7, 27. doi: 10.3389/fped.2019.00027, PMID: 30805325 PMC6378289

[ref5] Amiano N. O. Morelli M. P. Pellegrini J. M. Tateosian N. L. Rolandelli A. Seery V.. (2020). IFN-γ and IgG responses to *Mycobacterium tuberculosis* latency antigen Rv2626c differentiate remote from recent tuberculosis infection. Sci. Rep. 10:7472. doi: 10.1038/s41598-020-64428-z 32366931 PMC7198533

[ref6] Andersen P. Munk M. E. Pollock J. M. Doherty T. M. (2000). Specific immune-based diagnosis of tuberculosis. Lancet 356, 1099–1104. doi: 10.1016/S0140-6736(00)02742-2, PMID: 11009160

[ref7] Coppola M. Villar-Hernández R. van Meijgaarden K. E. Latorre I. Muriel Moreno B. Garcia-Garcia E.. (2020). Cell-mediated immune responses to *in vivo*-expressed and stage-specific *Mycobacterium tuberculosis* antigens in latent and active tuberculosis across different age groups. Front. Immunol. 11:103. doi: 10.3389/fimmu.2020.00103, PMID: 32117257 PMC7026259

[ref8] Cossarizza A. Chang H. D. Radbruch A. Acs A. Adam D. Adam-Klages S.. (2019). Guidelines for the use of flow cytometry and cell sorting in immunological studies (second edition). Eur. J. Immunol. 49, 1457–1973. doi: 10.1002/eji.201970107, PMID: 31633216 PMC7350392

[ref9] Domínguez J. Ruiz-Manzano J. De Souza-Galvão M. Latorre I. Milà C. Blanco S.. (2008). Comparison of two commercially available gamma interferon blood tests for immunodiagnosis of tuberculosis. Clin. Vaccine Immunol. 15, 168–171. doi: 10.1128/CVI.00364-07, PMID: 17978008 PMC2223867

[ref10] Drain P. K. Bajema K. L. Dowdy D. Dheda K. Naidoo K. Schumacher S. G.. (2018). Incipient and subclinical tuberculosis: A clinical review of early stages and progression of infection. Clin. Microbiol. Rev. 31, e00021–e000218. doi: 10.1128/CMR.00021-18, PMID: 30021818 PMC6148193

[ref11] Filipe-Santos O. Bustamante J. Chapgier A. Vogt G. de Beaucoudrey L. Feinberg J.. (2006). Inborn errors of IL-12/23- and IFN-γ-mediated immunity: molecular, cellular, and clinical features. Semin. Immunol. 18, 347–361. doi: 10.1016/j.smim.2006.07.010, PMID: 16997570

[ref12] Furin J. Cox H. Pai M. (2019). Tuberculosis. Lancet 393, 1642–1656. doi: 10.1016/S0140-6736(19)30308-3, PMID: 30904262

[ref13] Gallegos A. M. van Heijst J. W. J. Samstein M. Su X. Pamer E. G. Glickman M. S. (2011). A gamma interferon independent mechanism of CD4 T cell mediated control of *M. tuberculosis* infection in vivo. PLoS Pathog. 7:e1002052. doi: 10.1371/journal.ppat.1002052, PMID: 21625591 PMC3098235

[ref47] Global Tuberculosis Report (2021). Geneva: World Health Organization.

[ref14] Goletti D. Delogu G. Matteelli A. Migliori G. B. (2022). The role of IGRA in the diagnosis of tuberculosis infection, differentiating from active tuberculosis, and decision making for initiating treatment or preventive therapy of tuberculosis infection. Int. J. Infect. Dis. doi: 10.1016/j.ijid.2022.02.047, PMID: [Epub ahead of print]. 35257904

[ref15] Goletti D. Lindestam Arlehamn C. S. Scriba T. J. Anthony R. Cirillo D. M. Alonzi T.. (2018). Can we predict tuberculosis cure? What tools are available? Eur. Respir. J. 52:1801089. doi: 10.1183/13993003.01089-2018, PMID: 30361242

[ref16] Halliday A. Whitworth H. Kottoor S. H. Niazi U. Menzies S. Kunst H.. (2017). Stratification of latent *mycobacterium tuberculosis* infection by cellular immune profiling. J. Infect. Dis. 215, 1480–1487. doi: 10.1093/infdis/jix107, PMID: 28329119 PMC5451604

[ref17] Harari A. Rozot V. Enders F. B. Perreau M. Stalder J. M. Nicod L. P.. (2011). Dominant TNF-α+ *Mycobacterium tuberculosis*-specific CD4+ T cell responses discriminate between latent infection and active disease. Nat. Med. 17, 372–376. doi: 10.1038/nm.2299, PMID: 21336285 PMC6570988

[ref18] Hartman W. R. Pelleymounter L. L. Moon I. Kalari K. Liu M. Wu T.-Y.. (2010). CD38 expression, function, and gene resequencing in a human lymphoblastoid cell line-based model system. Leuk. Lymphoma 51, 1315–1325. doi: 10.3109/10428194.2010.483299, PMID: 20470215 PMC2892000

[ref19] Hiza H. Hella J. Arbués A. Magani B. Sasamalo M. Gagneux S.. (2021). Case–control diagnostic accuracy study of a non-sputum CD38-based TAM-TB test from a single milliliter of blood. Sci. Rep. 11, 13190. doi: 10.1038/s41598-021-92596-z, PMID: 34162973 PMC8222251

[ref20] Hiza H. Hella J. Arbués A. Sasamalo M. Misana V. Fellay J.. (2022). CD38 expression by antigen-specific CD4 T cells is significantly restored 5 months after treatment initiation independently of sputum bacterial load at the time of tuberculosis diagnosis. Front. Med. 9:821776. doi: 10.3389/fmed.2022.821776, PMID: 35492319 PMC9051241

[ref21] Houben R. M. G. J. Dodd P. J. (2016). The global burden of latent tuberculosis infection: A re-estimation using mathematical modelling. PLoS Med. 13:e1002152. doi: 10.1371/journal.pmed.1002152, PMID: 27780211 PMC5079585

[ref22] Lalvani A. Pareek M. (2010). A 100 year update on diagnosis of tuberculosis infection. Br. Med. Bull. 93, 69–84. doi: 10.1093/bmb/ldp039, PMID: 19926636

[ref23] Latorre I. De Souza-Galvão M. Ruiz-Manzano J. Lacoma A. Prat C. Altet N.. (2010). Evaluating the non-tuberculous mycobacteria effect in the tuberculosis infection diagnosis. Eur. Respir. J. 35, 338–342. doi: 10.1183/09031936.00196608, PMID: 20123845

[ref24] Latorre I. Fernández-Sanmartín M. A. Muriel-Moreno B. Villar-Hernández R. Vila S. De Souza-Galvão M. L.. (2019). Study of CD27 and CCR4 markers on specific CD4+ T-cells as immune tools for active and latent tuberculosis management. Front. Immunol. 9:3094. doi: 10.3389/fimmu.2018.03094, PMID: 30687314 PMC6334476

[ref26] Luo Y. Xue Y. Mao L. Lin Q. Tang G. Song H.. (2021). Activation phenotype of *Mycobacterium tuberculosis*-specific CD4+ T cells promoting the discrimination between active tuberculosis and latent tuberculosis infection. Front. Immunol. 12, 721013. doi: 10.3389/fimmu.2021.721013, PMID: 34512645 PMC8426432

[ref27] Lyadova I. V. Oberdorf S. Kapina M. A. Apt A. S. Swain S. L. Sayles P. C. (2004). CD4 T cells producing IFN-γ in the lungs of mice challenged with mycobacteria express a CD27-negative phenotype. Clin. Exp. Immunol. 138, 21–29. doi: 10.1111/j.1365-2249.2004.02573.x, PMID: 15373901 PMC1809176

[ref28] Metcalfe J. Z. Cattamanchi A. McCulloch C. E. Lew J. D. Ha N. P. Graviss E. A. (2013). Test variability of the QuantiFERON-TB gold in-tube assay in clinical practice. Am. J. Respir. Crit. Care Med. 187, 206–211. doi: 10.1164/rccm.201203-0430OC, PMID: 23103734 PMC3570654

[ref29] Morgan J. Muskat K. Tippalagama R. Sette A. Burel J. Lindestam Arlehamn C. S. (2021). Classical CD4 T cells as the cornerstone of antimycobacterial immunity. Immunol. Rev. 301, 10–29. doi: 10.1111/imr.12963, PMID: 33751597 PMC8252593

[ref30] Mpande C. A. M. Musvosvi M. Rozot V. Mosito B. Reid T. D. Schreuder C.. (2021). Antigen-specific T-cell activation distinguishes between recent and remote tuberculosis infection. Am. J. Respir. Crit. Care Med. 203, 1556–1565. doi: 10.1164/rccm.202007-2686OC, PMID: 33406011 PMC8483229

[ref31] Musvosvi M. Duffy D. Filander E. Africa H. Mabwe S. Jaxa L.. (2018). T-cell biomarkers for diagnosis of tuberculosis: candidate evaluation by a simple whole blood assay for clinical translation. Eur. Respir. J. 51, 1800153. doi: 10.1183/13993003.00153-2018, PMID: 29567725

[ref32] Nikitina I. Y. Kondratuk N. A. Kosmiadi G. A. Amansahedov R. B. Vasilyeva I. A. Ganusov V. V.. (2012). Mtb-specific CD27 low CD4 t cells as markers of lung tissue destruction during pulmonary tuberculosis in humans. PLoS One 7:e43733. doi: 10.1371/journal.pone.0043733, PMID: 22937086 PMC3427145

[ref33] O’Garra A. Redford P. S. McNab F. W. Bloom C. I. Wilkinson R. J. Berry M. P. R. (2013). The immune response in tuberculosis. Annu. Rev. Immunol. 31, 475–527. doi: 10.1146/annurev-immunol-032712-095939, PMID: 23516984

[ref34] Pai M. Behr M. A. Dowdy D. Dheda K. Divangahi M. Boehme C. C.. (2016). Tuberculosis. Nat. Rev. Dis. Primers. 2, 16076. doi: 10.1038/nrdp.2016.76, PMID: 27784885

[ref35] Petruccioli E. Navarra A. Petrone L. Vanini V. Cuzzi G. Gualano G.. (2016). Use of several immunological markers to model the probability of active tuberculosis. Diagn. Microbiol. Infect. Dis. 86, 169–171. doi: 10.1016/j.diagmicrobio.2016.06.007, PMID: 27431433

[ref36] Petruccioli E. Petrone L. Vanini V. Cuzzi G. Navarra A. Gualano G.. (2015). Assessment of CD27 expression as a tool for active and latent tuberculosis diagnosis. J. Infect. 71, 526–533. doi: 10.1016/j.jinf.2015.07.009, PMID: 26253021

[ref37] Pollock K. M. Whitworth H. S. Montamat-Sicotte D. J. Grass L. Cooke G. S. Kapembwa M. S.. (2013). T-cell immunophenotyping distinguishes active from latent tuberculosis. J Infect Dis 208, 952–968. doi: 10.1093/infdis/jit265, PMID: 23966657 PMC3749005

[ref38] Portevin D. Moukambi F. Clowes P. Bauer A. Chachage M. Ntinginya N. E.. (2014). Assessment of the novel T-cell activation marker-tuberculosis assay for diagnosis of active tuberculosis in children: A prospective proof-of-concept study. Lancet Infect. Dis. 14, 931–938. doi: 10.1016/S1473-3099(14)70884-9, PMID: 25185458

[ref39] Riou C. Berkowitz N. Goliath R. Burgers W. A. Wilkinson R. J. (2017). Analysis of the phenotype of *Mycobacterium tuberculosis*-specific CD+ T cells to discriminate latent from active tuberculosis in HIV-uninfected and HIV-infected individuals. Front. Immunol. 8:968. doi: 10.3389/fimmu.2017.00968, PMID: 28848561 PMC5554366

[ref400] Saraiva D. P. Jacinto A. Borralho P. Braga S. Cabral M. G. (2018). HLA-DR in cytotoxic T lymphocytes predicts breast cancer patients’ response to neoadjuvant chemotherapy. Front Immunol. 9:2605. doi: 10.3389/fimmu.2018.02605, PMID: 30555458 PMC6282034

[ref40] Schiött Å. Lindstedt M. Johansson-Lindbom B. Roggen E. Borrebaeck C. A. K. (2004). CD27- CD4+ memory T cells define a differentiated memory population at both the functional and transcriptional levels. Immunology 113, 363–370. doi: 10.1111/j.1365-2567.2004.01974.x, PMID: 15500623 PMC1782577

[ref41] Seddon J. A. Paton J. Nademi Z. Keane D. Williams B. Williams A.. (2016). The impact of BCG vaccination on tuberculin skin test responses in children is age dependent: evidence to be considered when screening children for tuberculosis infection. Thorax 71, 932–939. doi: 10.1136/thoraxjnl-2015-207687, PMID: 27335104 PMC5036222

[ref42] Silveira-Mattos P. S. Barreto-Duarte B. Vasconcelos B. Fukutani K. F. Vinhaes C. L. Oliveira-De-Souza D.. (2020). Differential expression of activation markers by *Mycobacterium tuberculosis*-specific CD4+T cell distinguishes extrapulmonary from pulmonary tuberculosis and latent infection. Clin. Infect. Dis. 71, 1905–1911. doi: 10.1093/cid/ciz1070, PMID: 31665254 PMC8463092

[ref43] Soares A. Govender L. Hughes J. Mavakla W. de Kock M. Barnard C.. (2010). Novel application of Ki67 to quantify antigen-specific *in vitro* lymphoproliferation. J. Immunol. Methods 362, 43–50. doi: 10.1016/j.jim.2010.08.007, PMID: 20800066 PMC2989440

[ref44] Tippalagama R. Singhania A. Dubelko P. Lindestam Arlehamn C. S. Crinklaw A. Pomaznoy M.. (2021). HLA-DR marks recently divided antigen-specific effector CD4 T cells in active tuberculosis patients. J. Immunol. 207, 523–533. doi: 10.4049/jimmunol.2100011, PMID: 34193602 PMC8516689

[ref45] Veneri D. Ortolani R. Franchini M. Tridente G. Pizzolo G. Vella A. (2009). Expression of CD27 and CD23 on peripheral blood B lymphocytes in humans of different ages. Blood Transfus. 7, 29–34. doi: 10.2450/2008.0007-08, PMID: 19290077 PMC2652233

[ref46] Vickers M. A. Darboe F. Muefong C. N. Mbayo G. Barry A. Gindeh A.. (2020). Monitoring anti-tuberculosis treatment response using analysis of whole blood *Mycobacterium tuberculosis* specific T cell activation and functional markers. Front. Immunol. 11:572620. doi: 10.3389/fimmu.2020.572620, PMID: 33679684 PMC7931252

[ref230] Walzl R. McNerney R. du Plessis N. Bates M. McHugh T. D. Chegou N. N.. (2018). Tuberculosis: advances and challenges in development of new diagnostics and biomarkers. Lancet Infect. Dis. 18, e199–e210. doi: 10.1016/S1473-3099(18)30111-7, PMID: 29580818

[ref48] Xu F. Zhang H. Si X. Chen J. Chen Y. Cui X.. (2021). Assessment of CD27 expression on T-cells as a diagnostic and therapeutic tool for patients with smear-negative pulmonary tuberculosis. BMC Immunol. 22, 41. doi: 10.1186/s12865-021-00430-y, PMID: 34176483 PMC8237462

