# Supplementary figures and images for: Study of CD27, CD38, HLA-DR and Ki-67 immune profiles for the characterization of active tuberculosis, latent infection and end of treatment
Source: Front Microbiol. 2022 Jul 22;13:885312. doi: 10.3389/fmicb.2022.885312 (PMC9354672; doi:10.3389/fmicb.2022.885312)

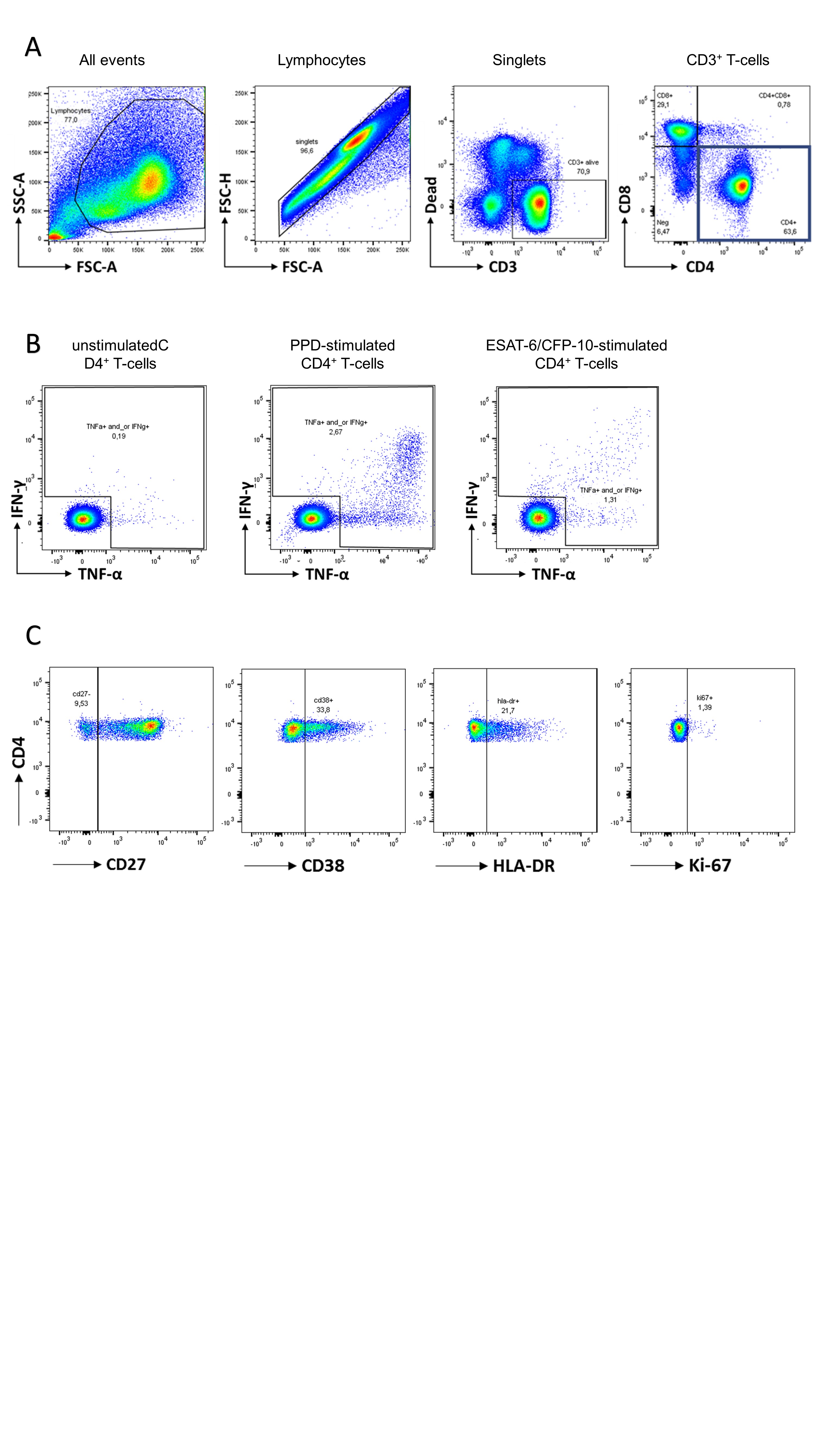

Supplement: Supplementary Figure 1 — Gating strategy used for CD4+ T-cells cytokine secretion and markers analysis. (A) After rough selection of lymphocytes, aggregated cells were taken off by gating on the diagonal that appears with Forward-Scatter (height; FSC-H) vs. Forward-Scatter (area; FSC-A) dot plot. CD4+ T-cells were gated from a parent population of alive CD3+ T-cells. (B) Mtb-specific population were selected from parent population CD4+ T-cells and gated based on the expression of TNF-α+ and/or IFN-γ+, from which CD27/CD38/HLA-DR/Ki67 expression was studied. The panels are representative of populations found without stimulation (left) and after PPD (middle) and ESAT-6/CFP-10 (right) stimulation. (C) Panels showing representative examples of cell distribution according to each marker of the study (sample shown is from an LTBI individual after PPD stimulation). The gates were decided according to FMO (Fluorescence Minus One) controls. [file Image_1.JPEG]

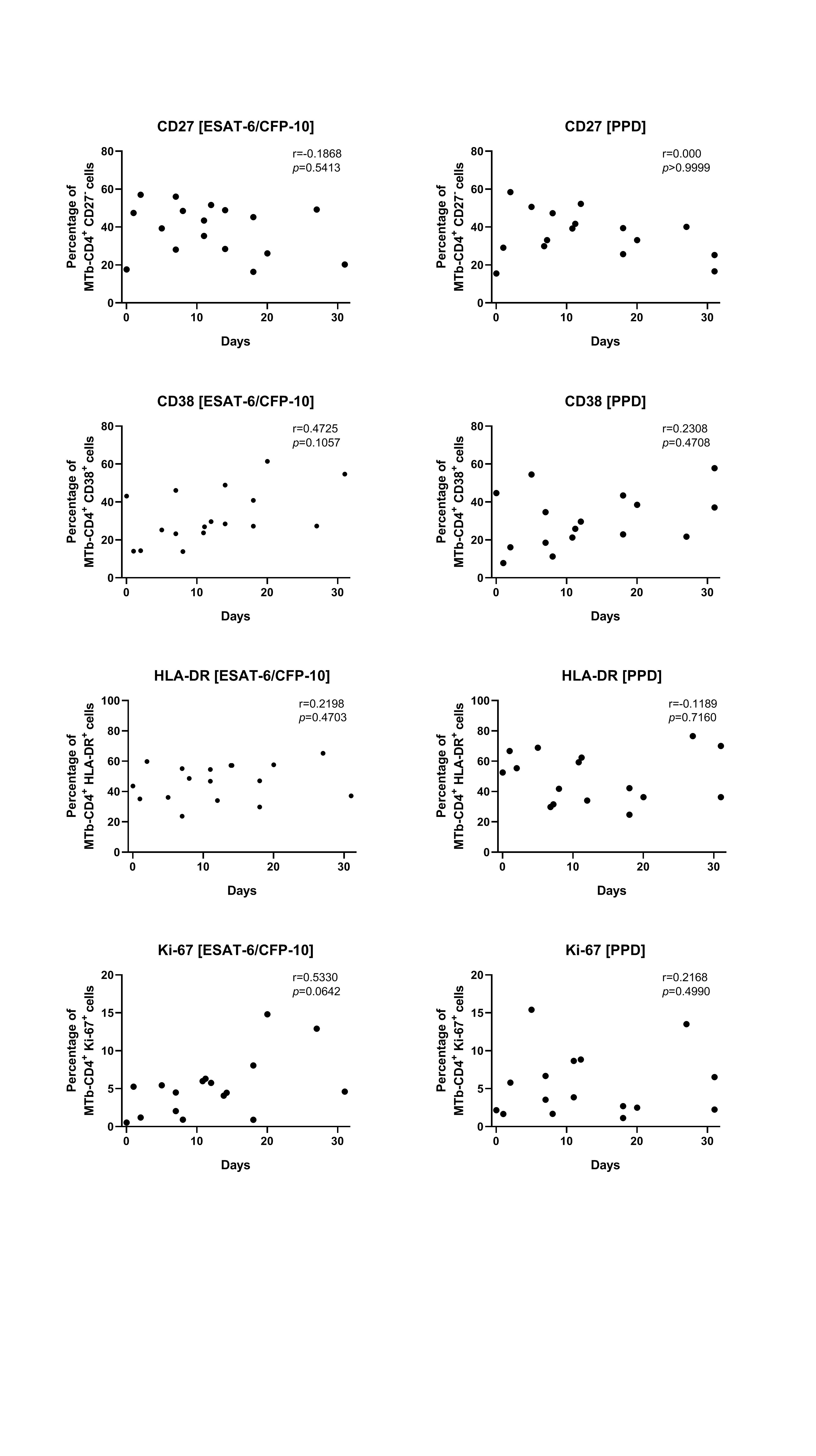

Supplement: Supplementary Figure 2 — Expression of markers in aTB patients according to the day of treatment within the first month. Correlation between the percentage of CD27−, CD38+, HLA-DR+ and Ki-67+ within TNF-α+ and/or IFN-γ+ CD4+ T-cells and the days after the start of the treatment during the first month in patients from the aTB group. [file Image_2.JPEG]

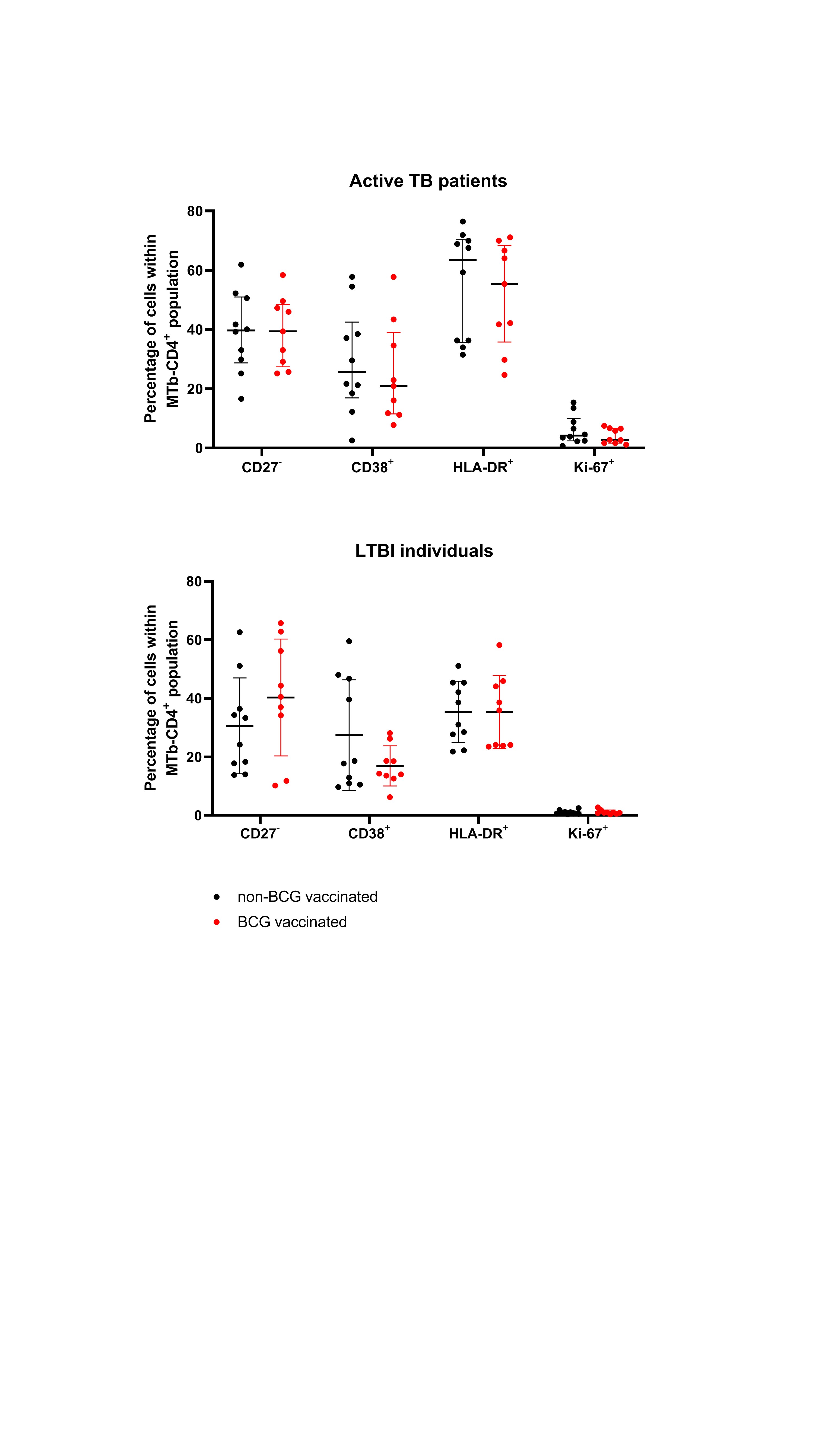

Supplement: Supplementary Figure 3 — BCG vaccination had no effect in the outcome measures of the analysis in PPD-stimulated samples. Percentage of CD27−, CD38+, HLA-DR+, and Ki67+ within TNF-α+ and/or IFN-γ+ CD4+ T-cells after stimulation with PPD. aTB patients (up) and LTBI individuals (down) are separated by their vaccination status of BCG. Differences between conditions were calculated using the Mann–Whitney U test. No indication of p value implies no significance. [file Image_3.JPEG]

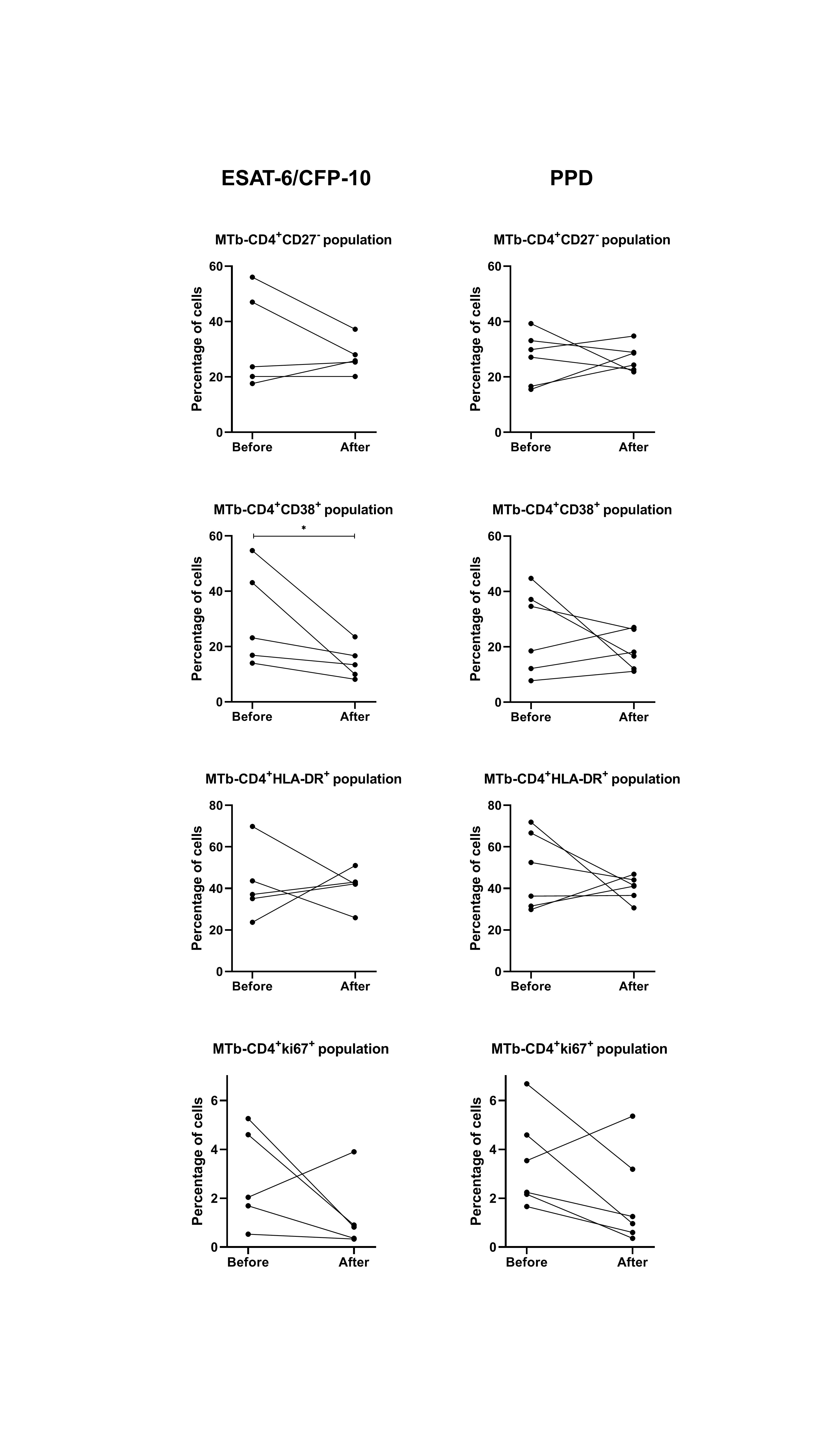

Supplement: Supplementary Figure 4 — Changes in CD27−, CD38+, HLA-DR+, and Ki67+ phenotypes of each monitored individual before and after treatment. Percentage of CD27−, CD38+, HLA-DR+, and Ki67+ within TNF-α+ and/or IFN-γ+ CD4+ T-cells after stimulation with ESAT-6/CFP-10 (left) or PPD (right) exclusively for the aTB patients that were monitored before (≤1 month) and after (≥6 months) anti-TB therapy. Because of exclusion from one of the points from both ends due to analysis restrictions, only patients with both samples positive for the stimulants are shown. Differences between conditions were calculated using the Wilcoxon signed rank paired test. *p < 0.05. No indication of p value implies non significance. [file Image_4.JPEG]

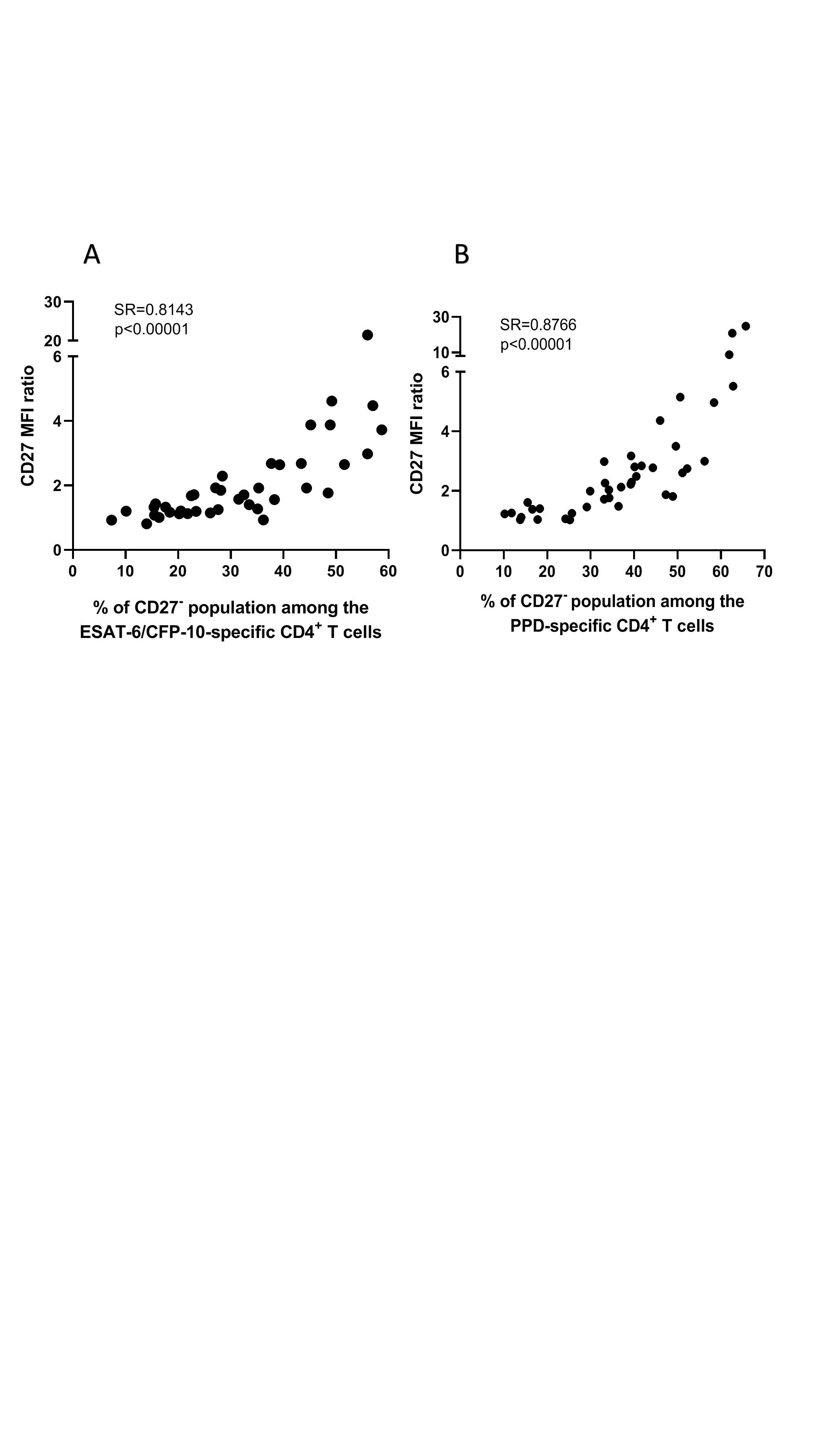

Supplement: Supplementary Figure 5 — Strong correlation between the percentage of CD27− in TNF-α+ and/or IFN-γ+ CD4+ T-cells and the CD27 MFI ratio after ESAT-6/CFP-10 (left) and PPD (right) stimulation. Correlation was calculated using the two-tailed non-parametric Spearman test. *p < 0.05, **p < 0.01, ****p < 0.0001. aTB, active TB; LTBI, latent tuberculosis infection; eTrt, aTB patients after anti-TB treatment. [file Image_5.JPEG]

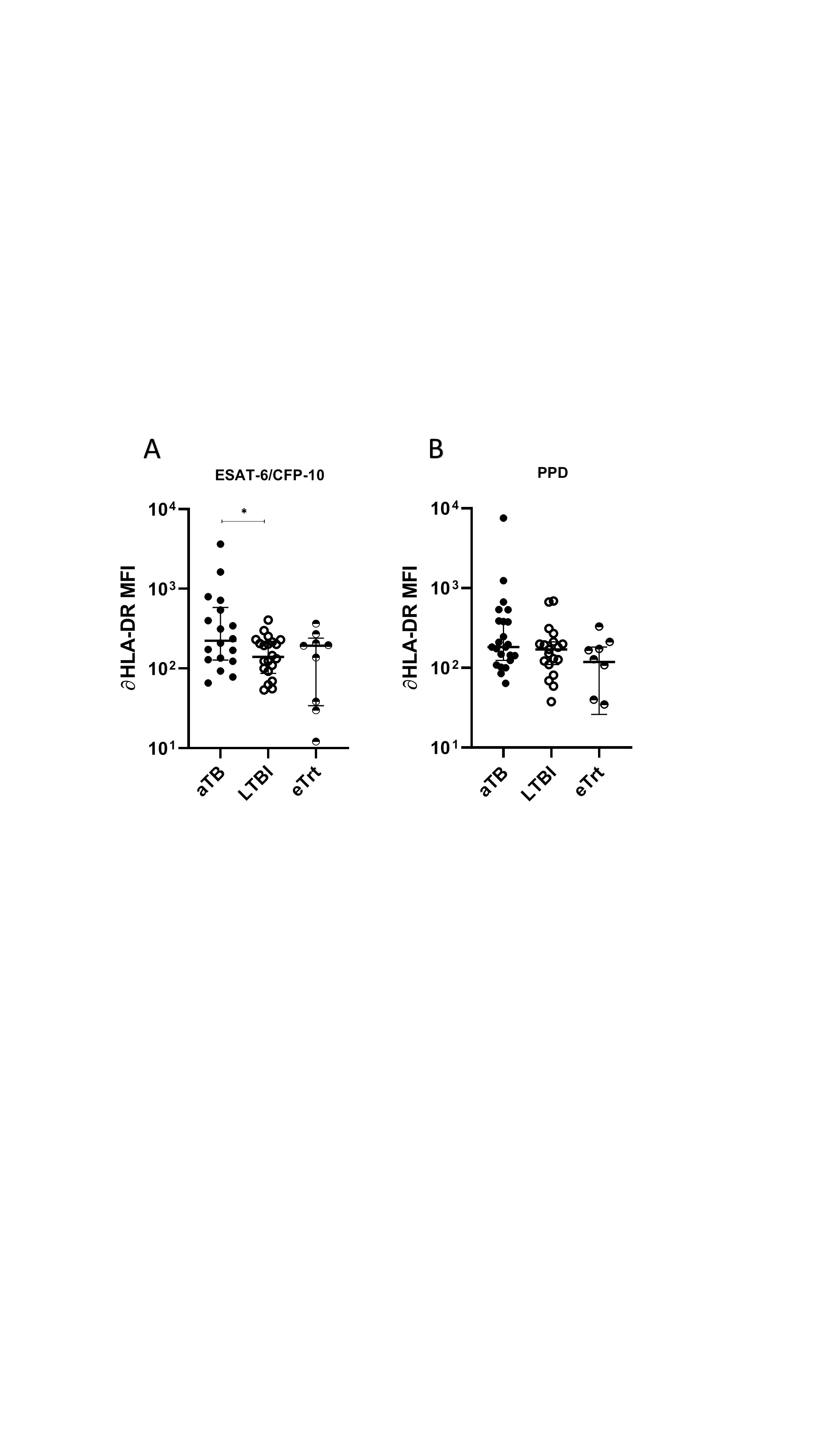

Supplement: Supplementary Figure 6 — ΔHLA-DR MFI of each respective group. ΔHLA-DR median fluorescent intensity (MFI) was calculated as suggested by Mpande et al. The numbers result from subtracting the HLA-DR MFI on IFN-γ+ and/or TNF+ CD3 + cells to the HLA-DR MFI on total CD3+ cells. (A) ΔHLA-DR MFI after ESAT-6/CFP10 or (B) PPD stimulation in patients with active TB in the beginning and end of treatment as well as LTBI individuals. Data plotted with median and interquartile range. Differences between aTB and LTBI conditions were calculated using the two-tailed Mann–Whitney U-test. Differences between aTB and eTrt groups were calculated using a mixed statistical model controlling repeated measures on logit-transformed data. *p < 0.05. [file Image_6.JPEG]

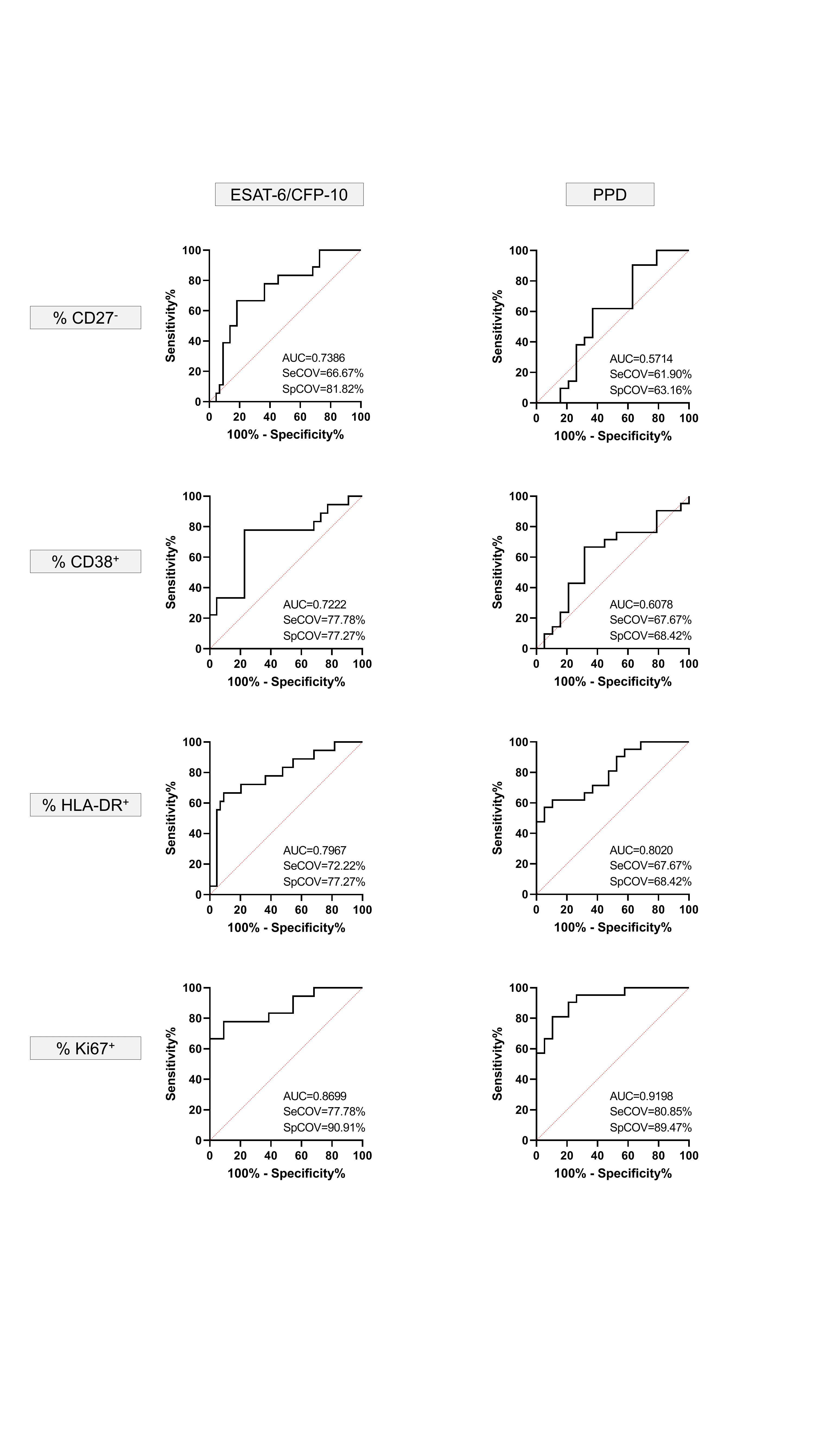

Supplement: Supplementary Figure 7 — Area under the receiver operating characteristic curve (AUROC) showing performance of CFP-10/ESAT-6–specific (left) and PPD-specific (right) TNF-α+ and/or IFN-γ+ CD4+ T-cells to discriminate between aTB and LTBI individuals for every marker studied in the paper. Se, sensitivity; Sp, specificity. [file Image_7.PNG]
