# Supplementary material for: Study of CD27, CD38, HLA-DR and Ki-67 immune profiles for the characterization of active tuberculosis, latent infection and end of treatment
Source: Front Microbiol. 2022 Jul 22;13:885312. doi: 10.3389/fmicb.2022.885312 (PMC9354672; doi:10.3389/fmicb.2022.885312)
Supplement: Supplementary file 8 [file Table_1.DOCX]

| **Antibody** | **Clone** | **Conjugate** | **Concentration** | **Company** |
| --- | --- | --- | --- | --- |
| **Mouse anti-human CD3** | SK7 | PerCP | 1:40 | BioLegend |
| **Mouse anti-human CD4** | SK3 | BV786 | 1:20 | BD Horizon™ |
| **Mouse anti-human CD8** | SK1 | BV510 | 1:40 | BioLegend |
| **Mouse anti-human CD27** | L128 | BV605 | 1:10 | BD Horizon™ |
| **Mouse anti-human CD38** | HIT2 | PE | 1:2.5 | BD Pharmingen™ |
| **Mouse anti-human HLA-DR** | G46-6 | BV421 | 1:20 | BD Horizon™ |
| **Anti-human IFN-γ** | B27 | APC | 1:100 | BD Pharmingen™ |
| **Anti-Mouse/Rat Ki-67** | SolA15 | FITC | 1:40 | ThermoFisher |
| **Mouse anti-human TNF**  **LIVE/DEAD Fixable Near-IR Dead Cell** | MAb11  - | PECy7  - | 1:200  1:10000 | BD Pharmingen™  ThermoFisher |

**Supplementary Table 1**. List of conjugated antibodies used for the flow-cytometry assay, with details on the clone species, concentration and origin.
